# Audiovestibular Dysfunction Related to Long COVID-19 Syndrome: A Systematic Review of Characteristics, Pathophysiology, Diagnosis, and Management

**DOI:** 10.3390/ijms27031417

**Published:** 2026-01-30

**Authors:** Jiann-Jy Chen, Chih-Wei Hsu, Hung-Yu Wang, Brendon Stubbs, Tien-Yu Chen, Chih-Sung Liang, Yen-Wen Chen, Bing-Syuan Zeng, Ping-Tao Tseng

**Affiliations:** 1 Prospect Clinic for Otorhinolaryngology & Neurology, Kaohsiung 811, Taiwan; jiannjy@yahoo.com.tw (J.-J.C.); kevinachen0527@gmail.com (Y.-W.C.); 2 Department of Otorhinolaryngology, E-Da Cancer Hospital, I-Shou University, Kaohsiung 824, Taiwan; 3 Department of Psychiatry, Kaohsiung Chang Gung Memorial Hospital and Chang Gung University College of Medicine, Kaohsiung 833, Taiwan; harwicacademia@gmail.com; 4 Kaohsiung Municipal Kai-Syuan Psychiatric Hospital, Kaohsiung 802, Taiwan; hywang1975@gmail.com; 5 Department of Psychological Medicine, Institute of Psychiatry, Psychology and Neuroscience, King’s College London, London WC2R 2LS, UK; brendon.stubbs@kcl.ac.uk; 6 Department of Sport, University of Vienna, 1090 Vienna, Austria; 7 Department of Psychiatry, Tri-Service General Hospital, School of Medicine, National Defense Medical University, Taipei 114, Taiwan; verducciwol@gmail.com; 8 Department of Psychiatry, College of Medicine, National Defense Medical University, Taipei 114, Taiwan; 9 Department of Psychiatry, Beitou Branch, Tri-Service General Hospital, School of Medicine, National Defense Medical University, Taipei 114, Taiwan; lcsyfw@gmail.com; 10 Department of Psychiatry, National Defense Medical University, Taipei 114, Taiwan; 11 Department of Internal Medicine, E-Da Cancer Hospital, I-Shou University, Kaohsiung 824, Taiwan; 12 Institute of Biomedical Sciences, National Sun Yat-Sen University, Kaohsiung 804, Taiwan; 13 Institute of Precision Medicine, National Sun Yat-Sen University, Kaohsiung 804, Taiwan

**Keywords:** long COVID-19 syndrome, cochleopathy, vestibular, sensorineural hearing loss, treatment

## Abstract

Long COVID-19 syndrome (or so-called post-COVID-19) is indicated by miscellaneous symptoms, usually starting 3 months from the COVID-19 infection and lasting for at least 2 months, which cannot be explained by an alternative diagnosis. There has been more and more reports addressing the audiovestibular dysfunction related to long COVID-19 syndrome. Emerging evidence suggests that the linkage between audiovestibular dysfunction and long COVID-19 syndrome might rely on (a) direct inner ear system damage related to viral invasion and consequent inflammation, (b) micro thromboembolic events, which might result from the COVID-19-induced autoimmune reaction against endothelial cells, and consequent transient-ischemia and hypoxia of the auditory pathways, (c) the disturbed nerve conduction in vestibulocochlear nerves due to viral invasion, and finally (d) altered auditory cortex function, either imbalanced central gain or neurotransmitter disturbance. However, most of the aforementioned mechanism remained hypothetic and still needed further studies to approve or refute. This systematic review synthesizes current evidence on the characteristics, pathophysiology, diagnostic approaches, and management of audiovestibular dysfunction related to long COVID-19 syndrome. Literature searches across PubMed, Embase, ClinicalKey, Web of Science, and ScienceDirect (up to 15 December 2025) were conducted in accordance with PRISMA guidelines. Through this systematic review, we provided a schematic diagram of the physiopathology of long COVID-19 syndrome-related audiovestibular dysfunction. Further, we summarized the currently available diagnostic tools to explore the audiovestibular function in such patients. The currently available treatment, either pharmacotherapy or nonpharmacotherapy, mainly tackles idiopathic audiovestibular dysfunction but not specifically long COVID-19 syndrome-related audiovestibular dysfunction. Timely recognition and intervention may prevent progression to permanent hearing loss or vestibular disability, improving quality of life. Trial registration: PROSPERO CRD420251265741.

## 1. Introduction

It has been 6 years since the first breakout of COVID-19. Beyond the respiratory system damage, there were more and more case reports addressing the audiovestibular dysfunction related to acute COVID-19 infection [1,2,3]. In a recent report by AlJasser and colleagues, the authors noticed that about 8% of cases complained of deterioration in hearing with/without tinnitus after COVID-19 infection [4]. Further, the subjects with COVID-19 infection had significant rates of vertigo compared to controls (5% versus 1%) [4]. Among all the audiovestibular symptoms related to COVID-19 infection, tinnitus accounted for the most prevalent (39%), followed by hearing loss (11%) [5]. Similar association was also detected in other reports, which revealed statistically significant differences in outcomes of hearing loss (3.1%) and tinnitus (4.5%) [6]. The presentation of audiovestibular dysfunction might appear either alone or in combination [7]. A histopathologic report focusing on temporal bone and inner ear tissue in both animals or humans revealed that COVID-19 could enter inner ear cells and result in consequent destruction via the mediation of angiotensin-converting enzyme 2 receptor, transmembrane protease serine 2, and FURIN cofactors, which were the key factors allowing for virus entry [8]. Although these audiovestibular symptoms related to COVID-19 infection might have subsided quickly (around 2–8 days) [9], there was still a great portion of subjects maintaining symptoms of audiovestibular dysfunction from months to years after, which is the so-called long COVID-19 period [10].

Long COVID-19 syndrome (or so-called post-COVID-19 [11,12]) indicated miscellaneous symptoms, usually starting 3 months from the COVID-19 infection and lasting for at least 2 months, which cannot be explained by an alternative diagnosis [13]. Several non-scientific terms had been developed to describe the neurologic presentation of long COVID-19 syndrome, such as long haulers [14] and brain fog [15]. The presence of long COVID-19 syndrome might not necessarily be dependent on the severity of the original COVID-19 infection [16]. In a large scale survey, about 12.7% patients had long COVID-19 syndrome 90–150 days (13–21 weeks) after COVID-19 infection [17]. In addition to symptomatic hearing loss related to COVID-19 infection, high-frequency hearing loss was also noted in asymptomatic subjects [18], while another report revealed insignificant findings [19]. Research has demonstrated that the infection of COVID-19 can lead to worse middle–high-frequency auditory thresholds [20] and abnormal findings in auditory brainstem response and transient evoked otoacoustic emissions [21] in the acute infection phase. While audiometry dysfunction caused by long COVID-19 may not trouble all patients [22], a certain number of individuals (approximately 1.99%) continue to experience hearing impairment about 6 months after COVID-19 infection [23]. On the other hand, in some cases, although some patients might not have any COVID-19 related symptoms, the high-frequency pure-tone thresholds [24] and transient evoked otoacoustic emissions amplitudes might be impaired [25] in subjects with positive test results for COVID-19.

In addition to audiometry dysfunction related to long COVID-19 syndrome, vestibular dysfunction was another important consequence in such patients. Specifically, around 34% subjects suffered vestibular symptoms (such as vertigo and unsteadiness) after acute COVID-19 infection [23]. These vestibular symptoms persisted continuously for 6 months after COVID-19 infection in about 3.99% of patients [23]. In addition to the impaired hearing threshold, the ability to discriminate words was also deteriorated in subjects with long COVID-19 syndrome [26].

Based on the fact that audiovestibular complications are highly associated with major psychiatric diseases [27], the exploration of the physiopathology and management against audiovestibular complications related to long COVID-19 syndrome would be highly clinically relevant.

Despite these insights, evidence of long COVID-19 syndrome’s audiovestibular effects is sparse, limiting clinical management. This systematic review aims to synthesize current knowledge on the characteristics, pathophysiology, diagnostic approaches, and treatment of audiovestibular dysfunction in patients with long COVID-19 syndrome, providing clinicians with evidence to guide practice and future research.

## 2. Methods and Materials

### 2.1. Study Design and Registration

**Figure 1:**
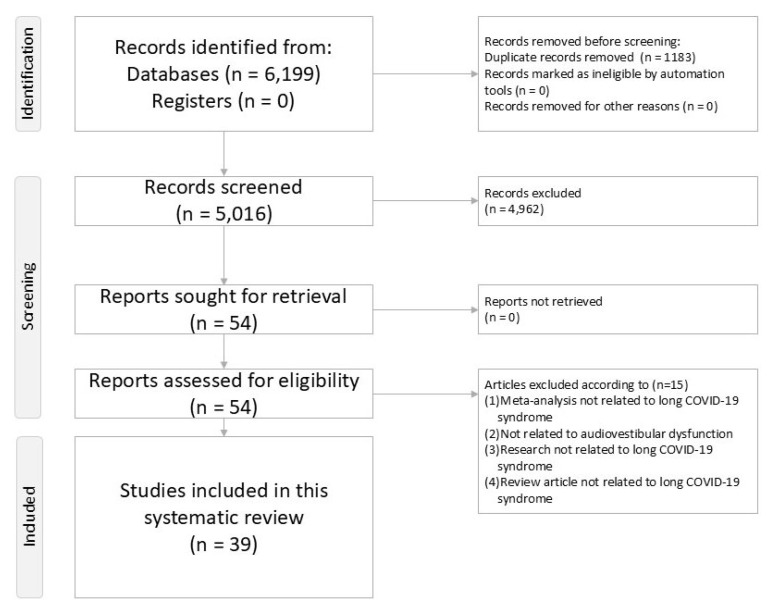
PRISMA2020 flowchart illustrating the procedure of the current systematic review.

This systematic review adhered to the Preferred Reporting Items for Systematic Reviews and Meta-Analyses (PRISMA) guidelines [28] and was registered with PROSPERO (CRD420251265741). A PRISMA checklist is provided (Table S1) and study selection is illustrated (Figure 1).

### 2.2. Search Strategy

We searched PubMed, Embase, ClinicalKey, Web of Science, and ScienceDirect from inception to 15 December 2025, using keywords and MeSH terms related to long COVID-19 syndrome and audiovestibular dysfunction (Table S2). Manual searches of reference lists from included studies and relevant reviews supplemented the electronic search. No language or publication date restrictions were applied. Corresponding authors were contacted for additional data when necessary.

### 2.3. Eligibility Criteria

Inclusion criteria were (1) studies addressing characteristics, pathophysiology, diagnosis, or treatment of audiovestibular dysfunction in patients, either adults or children, with long COVID-19 syndrome; (2) study designs including case reports, case series, observational studies, case–control studies, or randomized controlled trials; (3) studies involving patients diagnosed with long COVID-19 syndrome. The definition of long COVID-19 was defined according to a previous review article by Greenhalgh and colleagues [29].

Exclusion criteria were (1) studies unrelated to audiovestibular dysfunction characteristics, pathophysiology, diagnosis, or treatment; and (2) animal studies.

Excluded articles are listed in Table S3.

### 2.4. Screening and Selection

Two authors (PT Tseng, YW Chen) independently screened titles and abstracts from all databases, followed by full-text review of eligible articles. Duplicates were removed manually using reference management software. Discrepancies were resolved through discussion or consultation with a third author (JJ Chen).

### 2.5. Data Extraction

Two authors (PT Tseng, YW Chen) independently extracted data on study characteristics (e.g., design, sample size), patient demographics, audiovestibular dysfunction characteristics, pathophysiology, diagnostic methods, and treatments from full texts. Discrepancies were resolved through consensus.

### 2.6. Quality Assessment

Study quality was independently assessed by PT Tseng and YW Chen using the Newcastle–Ottawa Scale for non-randomized studies [30] or the Cochrane Risk of Bias tool for randomized trials, if applicable (Table S4). Discrepancies were resolved through discussion. Quality scores informed narrative synthesis but did not influence inclusion.

### 2.7. Data Synthesis

Data were synthesized narratively due to anticipated heterogeneity in study designs and outcomes. Findings were categorized by characteristics, pathophysiology, diagnosis, and treatment of audiovestibular dysfunction in long COVID-19 syndrome. Quantitative meta-analysis was not planned due to expected variability in study methodologies and reporting.

## 3. Results

After excluding 15 reports (Table S3), our search (up to 15 December 2025) included 39 reports. Study characteristics and quality assessments are detailed in Tables S4 and S5.

### 3.1. Epidemiology

The prevalence of audiovestibular symptoms varied across the COVID-19 infectious course. Specifically, in acute infection phase, the prevalence of auditory and vestibular symptoms were 21.9% and 34.9%, respectively [23]. There were 1.99% and 3.99% of patients who still had persistent auditory and vestibular symptoms at 6 months after COVID-19 infection [23]. The most frequently reported auditory discomfort included hearing loss, tinnitus, aural fullness, and earache [31]. Among them, hearing loss and tinnitus accounted for about 15% of cases [32]. On the other hand, vertigo and unsteadiness were the most frequently mentioned vestibular symptoms [23]. In another large-scale online-survey study, the authors noticed that, among long COVID-19 subjects after a mean period of 43.2 weeks from initial infection, 60% of patients were reporting the presence of vertigo and 30% tinnitus [33]. Further, about 1/5 of patients with tinnitus/vertigo considered their symptoms as severe. Similar findings could also be found in other reports [34,35]. In the report by Pazdro-Zastawny and colleagues, vertigo of central origin was the main presentation of vertigo in subjects with long COVID-19 syndrome, whereas peripheral or mixed vestibular symptoms accounted for a lesser proportion [36]. Saniasiaya had reported two cases with presentation of vestibular migraine related to long COVID-19 syndrome [37]. Female subjects might be more prone to such sensory impairment than male ones [38]. Furthermore, this audiovestibular dysfunction not only happened in adult subjects, but also occurred in pediatric cases with long COVID-19 syndrome [39]. However, as addressed before, since the audiovestibular impairment might be detected in asymptomatic subjects [18], the prevalence rate of audiovestibular dysfunction in long COVID-19 syndrome might be underestimated.

### 3.2. Pathophysiology: (Table 1)

In the report by Bhatta and colleagues, the authors noticed that the finding of conductive hearing loss was corelated with the nasopharyngeal inflammation of COVID-19 infection [31]. In the report by Davies and colleagues, the authors noticed that neuropilin-1 was a COVID-19 infection mediator, especially in the neurologic system [40]. Further, as addressed before, COVID-19 would enter neuron cells of vestibulocochlear nerves via the mediation of angiotensin-converting enzyme 2, transmembrane protease serine 2, and FURIN cofactors (so-called COVID-19 neurotropism), leading to direct damage to the vestibulocochlear nerve and consequent audiovestibular dysfunction [41]. There was another hypothetic etiology toward vestibulocochlear damage other than an inflammatory process. Specifically, micro thromboembolic events, which might result from the COVID-19-induced autoimmune reaction against endothelial cells [42], and consequent transient-ischemia and hypoxia of the auditory pathways might also be responsible for persistent audiological symptoms in long COVID-19 patients [43].

**Table 1:** Summary of pathophysiology.

Mechanisms	Description
Established mechanisms	Hearing loss corelated with the nasopharyngeal inflammation induced by COVID-19 infection
	COVID-19 entered neuron cells via the mediation of ACE2, transmembrane protease serine 2, and FURIN cofactors
	COVID-19 virus spreads up to the olfactory bulb passing through the olfactory epithelium and lamina cribrosa
	Reduction in cortical GABA levels in patients with long COVID-19 syndrome
	COVID-19 results in pathological changes with features of disseminated encephalomyelitis
Hypothesized mechanisms	Neuropilin-1 worked as a COVID-19 infection mediator in the neurologic system
	COVID-19 might lead to direct damage to the vestibulocochlear nerve and consequent audiovestibular dysfunction
	Micro thromboembolic events related to COVID-19-induced autoimmune reaction against endothelial cells
	COVID-19 infection might affect the auditory signal transduction system by damaging the central hearing system
	COVID-19 virus spread up to the auditory cortex

In addition to direct inflammatory damage related to COVID-19 infection, the changes in auditory brainstem-evoked potentials tests may also reflect the fact that COVID-19 infection might affect the auditory signal transduction system by damaging the central hearing system (i.e., the brainstem) [44]. Specifically, the COVID-19 infection would result in vestibulocochlear nerve damage, which might persist despite being recovered from a COVID-19 infection episode [45]. On the other hand, although there was no direct evidence, the spread of the COVID-19 virus up to the auditory cortex might be another hypothetic etiology [43]. Specifically, the COVID-19 virus has been proven to be able to spread up to the olfactory bulb passing through the olfactory epithelium and lamina cribrosa [46], which are located just next to the auditory cortex [43], leading to neuroinflammation and consequent hearing symptoms.

In addition to middle/inner ear damage and nerve conduction impairment, the alteration of neurotransmitters in the central nervous system also play an important role in the formation of tinnitus related to long COVID-19 syndrome. Specifically, the reduction in cortical GABA levels in patients with long COVID-19 syndrome have been addressed [47]. The infection of COVID-19 can result in pathological changes, with features of disseminated encephalomyelitis in some cases [48]. In addition to direct damage related to COVID-19 infection, the previous research has demonstrated that the brainstem is also highly prone to pathological immune or vascular inflammation, which has also been observed in autopsy of COVID-19 cases [49]. In addition, through various damage pathways, the COVID-19 infection can lead to reduced Intracortical GABAergic inhibition which might consequently enhance the auditory central gain observed in long COVID-19 syndrome, which might last from 9 to 13 weeks [50] to 6 months [47].

### 3.3. Diagnostic Approaches

Although pure tone audiometry was the most frequently used first-line tool to detect hearing dysfunction in clinical practice, the applicability of pure tone audiometry in subjects with long COVID-19 syndrome might not be suitable. Specifically, in the report by Boboshko and colleagues, the authors did not detect significant changes in pure tone audiometry before/after COVID-19 infection despite patients’ subjective hearing dysfunction [26]. Regardless of the presence of COVID-19 symptoms, there was no significant differences by pure tone audiometry in long COVID-19 patients [51]. Similar findings could be found in another report which compared the pure tone audiometry before and 1 year after COVID-19 infection and revealed insignificant differences [52]. Therefore, advanced diagnostic tools should be considered in the approach toward subjects with long COVID-19 syndrome.

In the report by Bhatta [31], the authors noticed that type B and type C curves (i.e., middle ear dysfunction) in impedance audiometry could be detected in 1.15–5.1% patients with COVID-19 infection. Furthermore, the dysfunction impedance audiometry in most of these patients did not recover after 3 months post COVID-19 infection [31]. In the report by Öztürk and colleagues, there was a significantly increased threshold of extended high-frequency in audiometry tests accompanied with lower amplitudes in transient evoked otoacoustic emissions and distortion product otoacoustic emission [53]. Similarly, in the report by Gedik and colleagues, the auditory brainstem responses demonstrated increased III–V interpeak latencies in patients with long COVID-19 syndrome [21]. Further, the middle ear inflammation related to COVID-19 infection led to absent stapes reflex in almost 20% of post-COVID-19 patients [54]. In the report by Hamdy and colleagues, the authors noticed that the P300 (a cortical cognitive auditory evoked potential) latency was significantly different in participants with long COVID-19 compared with controls, suggesting slowed higher-order auditory–cognitive processing [45]. Consistently, long COVID-19 participants showed poorer speech intelligibility in quiet/noise and lower dichotic digits test performance, together with reduced MoCA scores, which may indicate involvement of central (cortical) auditory processing and cognition [26].

Regarding the diagnosis of vestibular dysfunction related to long COVID-19 syndrome, several traditional balance function tests might help in diagnosis of balance problems in such patients. For example, regarding peripheral vestibular hypofunction, Yılmaz and colleagues noticed that patients with long COVID-19 syndrome showed significantly impaired performance in the tests of vestibular evoked myogenic potentials and video head impulse test [55]. In addition, the direction-changing gaze-evoked nystagmus, vertical nystagmus, pursuit/saccade abnormal, and skew might be able to assess central vestibular disorder in such conditions. Although the results were promising, this diagnostic tool remained in the experimental stage. Furthermore, there was no conclusive evidence regarding the superiority of individual tests compared to the others. Therefore, further validation and correlation with the existing diagnostic tools are still in urgent need.

Regarding the image tool, the currently available routine image study, such as brain MRI (magnetic resonance imaging), could not provide sufficient power in the diagnosis of audiovestibular dysfunction in long COVID-19 syndrome [34].

Finally, although the diagnostic tool addressed above might help to detect the audiovestibular dysfunction in long COVID-19 patients, they could not distinguish the audiovestibular dysfunction as being COVID-19-related and non-COVID-19-related [56]. Therefore, the diagnosis of long COVID-19-related audiovestibular dysfunction still relied on history taking and exclusion of other causes after diagnostic proof-tested tools.

### 3.4. Treatment

First of all, there has not been trials directly investigating specific treatment against audiovestibular dysfunction related to long COVID-19 syndrome [57]. Although the prescription of steroid, via either oral, intravenous, or intratympanic route, might theoretically be effective in the management of inflammatory process related to COVID-19 injury [58], it did not always provide positive efficacy [59]. Kelleni proposed a potential benefit of metformin for the management of idiopathic or long COVID-19 tinnitus in a case report, but it did not specifically address long COVID-19 syndrome-related tinnitus [60]. Other treatments, such as hyperbaric oxygen therapy, mesoglican, monoclonal antibodies, and immunosuppressive therapy, might be considered to manage audiovestibular dysfunction related to long COVID-19 syndrome [61]. However, most of them were rooted in treatments for idiopathic audiovestibular dysfunction but not directly for long COVID-19 syndrome. In addition to pharmacological treatment, the traditional balance rehabilitation, such as vestibular physical therapy, could be beneficial for such patients with major balance dysfunction [62]. In addition to traditional balance function test, researchers have implanted the robotic device hunova to assist in functional sensorimotor assessment and rehabilitation in such patients [63]. Based on our previous network meta-analysis regarding management of vertigo, vestibular rehabilitation could provide superior efficacy toward chronic vertigo in comparison with simple medication treatment [64]. The balance rehabilitation, either at home or at a specific institution, could contribute significant improvements in balance function if continued for at least 3 months [65]. Therefore, an integrated management with a vestibular-centric approach, including first-line vestibular rehabilitation, cautious use of vestibular suppressants, and multidisciplinary balance approach, could be clinically appreciated for such patients with persistent symptoms.

In contrast, the prevention of re-infection of COVID-19 could be an important issue in the management of audiovestibular dysfunction related to long COVID-19 syndrome. Specifically, the infection of COVID-19 could exacerbate the severity of pre-existing audiovestibular dysfunction [66].

### 3.5. Prognosis

In the report by Niemczak and colleagues, the authors noticed that young adults may be more susceptible than older adults to the consequence of long COVID-19 syndrome with regard to central auditory processing [44]. In another large-scale report, the authors noticed that the severity of neurological symptoms during the acute stage of COVID-19 were associated with an increased likelihood of reporting hearing loss [67]. Furthermore, damage to the inner ear and the auditory pathway (i.e., vestibulocochlear nerve and central nervous system) could have long-lasting effects on the auditory system and on cognitive processing and attention [45]. Although nearly all patients recovered within six months [38], there was still some cases with long-lasting, persistent audiovestibular symptoms. In contrast, although most reports suggested that the audiovestibular dysfunction might gradually resolve along with the COVID-19 course, researchers noticed that, in the study by Hastie and colleagues, the prevalence of hearing and balance problems was significantly higher 12 months after COVID-19 infection than 6 months after [68].

## 4. Discussion

We noticed that the prevalence of audiovestibular dysfunction in long COVID-19 syndrome ranges widely across different studies. In particular, the prevalence in the report by Degen and colleagues was much higher than the others [33]. This difference might be associated with the relatively low prior COVID-19 vaccination rate in their study (about 80% subjects did not have prior COVID-19 vaccination) [33].

Along with the histopathologic evidence addressing the direct damage to inner ear tissue related to COVID-19 infection [8], the COVID-19 infection could also contribute to direct inner ear damage. In the report by Davies and colleagues, the authors noticed that neuropilin-1, a protein that serves as an important mediator for the integrity of cochlear function [69], was a COVID-19 infection mediator, especially in the neurologic system [40]. In addition to direct cochlear damage, the neuroinvasive and neurovirulent behavior of COVID-19 viruses toward nerve conduction in vestibulocochlear nerves might also explain the presence of abnormal auditory brainstem-evoked responses [70]. The degenerative vestibulocochlear nerve, either by an internal aging process or by external virulent damage, could amplify the “central gain” in the central nervous system [71], the effects of which could lead to a desynchronized signal in the patients’ brain [72] and lead to tinnitus and hyperacusis symptoms [73]. The linkage between tinnitus/hyperacusis and central auditory gain enhancement can be detected via electrophysiological measurements in animal models [74] and human studies [75]. Furthermore, the reduced GABA level in the cortex of subjects with long COVID-19 syndrome [47] might exacerbate the presentation of audiovestibular function imbalance in the central nervous system. In a functional neuroimage study, Isler and colleagues noticed that the reduced GABA levels had strong association with the formation of tinnitus in subjects without definite hearing loss [76]. Tinnitus, one of the most frequently reported auditory symptoms in long COVID-19 syndrome, was considered to be associated with overt auditory central gain [77] with or without middle/inner ear damage [73]. Evidence has demonstrated that tinnitus might happen in subjects with cochlear neuron degeneration [78] or central nervous neurodegeneration [79] despite normal hearing test results. Taken together, the delayed neural conduction in vestibulocochlear nerves and the increased central gain in the central nervous system could ultimately result in functional cortical processing disparities in long COVID-19 syndrome subjects [44].

One recent report by Sivagurunathan et al. revealed that COVID-19 infection could be associated with premature neuronal aging and consequent neurodegenerative diseases [80]. Neural degeneration, either primary or secondary, could result in reduced action potential amplitudes in electrocochleograms, which could lead to compensated central gain in tinnitus/hyperacusis patients [81]. As neurons were not readily regenerative, the COVID-19 related damage in the central nervous system might last for months to years, which is consistent with the features of long COVID-19 syndrome [82]. However, several mechanisms (e.g., cortical invasion, premature neuronal aging, GABA-mediated central gain) were still hypothesis-driven and needed future clarification.

Some might argue that the audiovestibular dysfunction in long COVID-19 syndrome might result from cognitive impairment due to the inflammatory process of COVID-19 (so-called brain fog) [83]. This argument mainly stems from the fact that the most frequently used first-line tool to detect audiovestibular dysfunction required highly attention-dependent work during examination. However, along with the implementation of diagnostic tools with less necessity for attention, researchers noticed that there was more and more evidence regarding the audiovestibular deficit in subjects with long COVID-19 syndrome. For example, the P300, a component of event-related potential, was proven to have a statistically significant association with the degrees of hearing loss despite potential bias by patients’ subjective attention [84]. The application of auditory brainstem response could also support the diagnosis of hearing loss in patients with long COVID-19 syndrome, in which case the results of audiometry testing may be argued to be biased due to the attention impairment related to brain fog from long COVID-19 syndrome.

### 4.1. Clinical Recommendations

As addressed before, there has not been direct treatment against audiovestibular dysfunction related to long COVID-19 syndrome. However, several treatment options for idiopathic audiovestibular dysfunction, which targeted similar physiopathology, might theoretically be beneficial to manage such problems, such as immune modulating therapy, non-invasive brain stimulation, and steroids [85,86,87,88]. In another aspect, although there had not been consensus regarding the red flag or timing for referral to an ENT doctor, we summarized some potential time points for referral consideration based on our previous report and clinical practice experience [89,90]. First, patients, either with or without previous history of COVID-19 infection, started to complain of audiovestibular symptoms, such as decreased hearing function, tinnitus, hyperacusis/hypoacusis, vertigo, and balance problems. Second, patients complain of unexplained neurologic symptoms, either with or without subjectively audiovestibular symptoms. Third, clinicians should consider ENT doctor referral if patients’ audiovestibular symptoms are less likely related to ototoxicity of a currently prescribed medication. Finally, although subjectively, clinicians should consider the option of ENT referral if their voice was frequently ignored by a specific patient. Similar with inner ear diseases related to other inflammatory processes [89,90,91,92,93,94], the audiovestibular dysfunction related to long COVID-19 syndrome might be manageable if detected early enough. However, delayed detection and management might lead to irreversible damage and consequent hearing loss and balance disorders.

### 4.2. Strengths and Limitations

This review’s strengths include adherence to PRISMA guidelines, comprehensive search strategies, and dual-author data extraction, providing a foundational synthesis despite limited evidence. However, the scarcity of direct long COVID-19 syndrome studies, especially studies investigating treatment, limits generalizability. The absence of histopathological data hinders mechanistic confirmation, and potential publication bias may overlook negative findings. Small sample sizes in case reports further constrain robustness. In addition, since children with COVID-19 always had minor symptoms, the audiovestibular dysfunction related to long COVID-19 syndrome in children has been ignored to date. Finally, although many audiovestibular tests have been proposed, there was insufficient evidence regarding the standardized diagnostic protocols, discordant patterns, sensitivity, and specificity of such tests. Specifically, to date, there has not been conclusive evidence regarding the superiority of specific vestibular assessment in long COVID-19.

## 5. Conclusions

**Figure 2:**
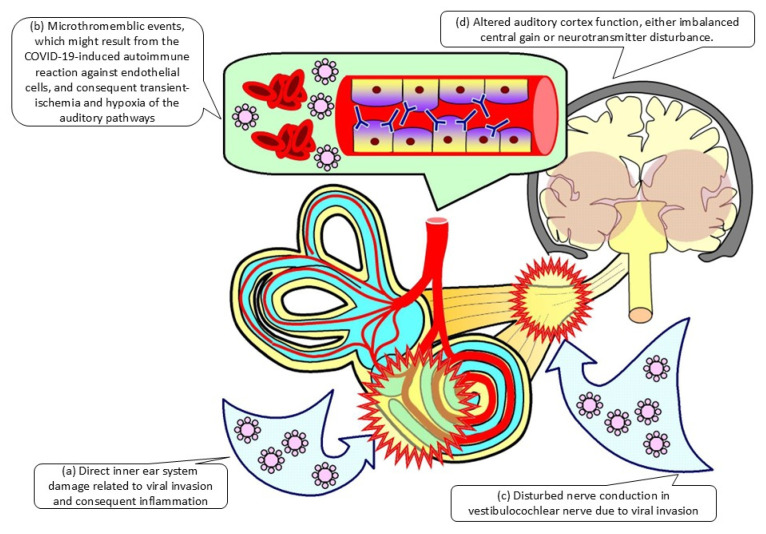
Schematic diagram of the hypothetical physiopathology of long COVID-19 syndrome in audiovestibular dysfunction. Figure 2 illustrates the hypothetical physiopathology of long COVID-19 syndrome-associated audiovestibular dysfunction. Overall, it consisted of several mechanisms, including (**a**) direct inner ear system damage related to viral invasion and consequent inflammation, (**b**) micro thromboembolic events, which might result from the COVID-19-induced autoimmune reaction against endothelial cells, and consequent transient-ischemia and hypoxia of the auditory pathways, (**c**) the disturbed nerve conduction in vestibulocochlear nerves due to viral invasion, and finally (**d**) altered auditory cortex function, either imbalanced central gain or neurotransmitter disturbance.

This review article has synthesized the current knowledge about audiovestibular dysfunction related to long COVID-19 syndrome. Figure 2 depicts a schematic diagram regarding the overall physiopathology of long COVID-19 syndrome associated with audiovestibular impairment. Audiovestibular dysfunction in long COVID-19 syndrome poses risks of long-term sensorineural hearing loss and vestibular impairment. Routine audiometric and vestibular screening is critical to detect early symptoms, enabling timely immunomodulatory interventions to prevent irreversible disability and enhance quality of life. Multidisciplinary care integrating virology, otolaryngology, and immunology is essential. Future research should prioritize prospective studies to establish targeted therapies and optimize clinical outcomes for long COVID-19 syndrome patients.

## Data Availability

No new data were created or analyzed in this study. These data were derived from the following resources available in the public domain: https://clinicaltrials.gov/.

## Supplementary Materials

The following supporting information can be downloaded at: https://www.mdpi.com/article/10.3390/ijms27031417/s1. References [2,6,7,8,10,11,14,20,21,23,24,26,28,31,32,33,34,35,36,37,38,39,40,41,42,43,44,45,46,47,48,49,50,51,52,53,54,55,56,57,58,59,60,61,62,63,66,67,68,87,89,90,91,92,93] are cited in the Supplementary Materials.
