# Supplementary material for: Audiovestibular Dysfunction Related to Long COVID-19 Syndrome: A Systematic Review of Characteristics, Pathophysiology, Diagnosis, and Management
Source: Int J Mol Sci. 2026 Jan 30;27(3):1417. doi: 10.3390/ijms27031417 (PMC12898127; doi:10.3390/ijms27031417)
Supplement: Supplementary file 1 [file ijms-27-01417-s001.zip › ijms-4092746-supplementary.pdf]

Table S1. PRISMA 2020 checklist of current systematic review.

Section and Topic	Item #	Checklist item	Page where item is reported
**TITLE**			
Title	1	Identify the report as a systematic review.	1
**ABSTRACT**			
Abstract	2	See the PRISMA 2020 for Abstracts checklist.	4
**INTRODUCTION**			
Rationale	3	Describe the rationale for the review in the context of existing knowledge.	6-7
Objectives	4	Provide an explicit statement of the objective(s) or question(s) the review addresses.	6-7
**METHODS**			
Eligibility criteria	5	Specify the inclusion and exclusion criteria for the review and how studies were grouped for the syntheses.	8-9
Information sources	6	Specify all databases, registers, websites, organisations, reference lists and other sources searched or consulted to identify studies. Specify the date when each source was last searched or consulted.	8-9
Search strategy	7	Present the full search strategies for all databases, registers and websites, including any filters and limits used.	8-9
Selection process	8	Specify the methods used to decide whether a study met the inclusion criteria of the review, including how many reviewers screened each record and each report retrieved, whether they worked independently, and if applicable, details of automation tools used in the process.	8-9
Data collection process	9	Specify the methods used to collect data from reports, including how many reviewers collected data from each report, whether they worked independently, any processes for obtaining or confirming data from study investigators, and if applicable, details of automation tools used in the process.	8-9
Data items	10a	List and define all outcomes for which data were sought. Specify whether all results that were compatible with each outcome domain in each study were sought (e.g. for all measures, time points, analyses), and if not, the methods used to decide which results to collect.	8-9
	10b	List and define all other variables for which data were sought (e.g. participant and intervention characteristics, funding sources). Describe any assumptions made about any missing or unclear information.	9-10
Study risk of bias assessment	11	Specify the methods used to assess risk of bias in the included studies, including details of the tool(s) used, how many reviewers assessed each study and whether they worked independently, and if applicable, details of automation tools used in the process.	9-10
Effect measures	12	Specify for each outcome the effect measure(s) (e.g. risk ratio, mean difference) used in the synthesis or presentation of results.	9-10
Synthesis methods	13a	Describe the processes used to decide which studies were eligible for each synthesis (e.g. tabulating the study intervention characteristics and comparing against the planned groups for each synthesis (item #5)).	Not done
	13b	Describe any methods required to prepare the data for presentation or synthesis, such as handling of missing summary statistics, or data conversions.	Not done
	13c	Describe any methods used to tabulate or visually display results of individual studies and syntheses.	Not done
	13d	Describe any methods used to synthesize results and provide a rationale for the choice(s). If meta-analysis was performed,	Not done

Section and Topic	Item #	Checklist item	Page where item is reported
		describe the model(s), method(s) to identify the presence and extent of statistical heterogeneity, and software package(s) used.	
	13e	Describe any methods used to explore possible causes of heterogeneity among study results (e.g. subgroup analysis, meta-regression).	Not done
	13f	Describe any sensitivity analyses conducted to assess robustness of the synthesized results.	Not done
Reporting bias assessment	14	Describe any methods used to assess risk of bias due to missing results in a synthesis (arising from reporting biases).	9-10
Certainty assessment	15	Describe any methods used to assess certainty (or confidence) in the body of evidence for an outcome.	9-10
**RESULTS**			
Study selection	16a	Describe the results of the search and selection process, from the number of records identified in the search to the number of studies included in the review, ideally using a flow diagram.	11-12
	16b	Cite studies that might appear to meet the inclusion criteria, but which were excluded, and explain why they were excluded.	11-12
Study characteristics	17	Cite each included study and present its characteristics.	11-12
Risk of bias in studies	18	Present assessments of risk of bias for each included study.	11-12
Results of individual studies	19	For all outcomes, present, for each study: (a) summary statistics for each group (where appropriate) and (b) an effect estimate and its precision (e.g. confidence/credible interval), ideally using structured tables or plots.	11-12
Results of syntheses	20a	For each synthesis, briefly summarise the characteristics and risk of bias among contributing studies.	Not done
	20b	Present results of all statistical syntheses conducted. If meta-analysis was done, present for each the summary estimate and its precision (e.g. confidence/credible interval) and measures of statistical heterogeneity. If comparing groups, describe the direction of the effect.	Not done
	20c	Present results of all investigations of possible causes of heterogeneity among study results.	Not done
	20d	Present results of all sensitivity analyses conducted to assess the robustness of the synthesized results.	Not done
Reporting biases	21	Present assessments of risk of bias due to missing results (arising from reporting biases) for each synthesis assessed.	13-14
Certainty of evidence	22	Present assessments of certainty (or confidence) in the body of evidence for each outcome assessed.	13-14
**DISCUSSION**			
Discussion	23a	Provide a general interpretation of the results in the context of other evidence.	16-18
	23b	Discuss any limitations of the evidence included in the review.	16-18
	23c	Discuss any limitations of the review processes used.	16-18
	23d	Discuss implications of the results for practice, policy, and future research.	19
**OTHER INFORMATION**			

Section and Topic	Item #	Checklist item	Page where item is reported
Registration and protocol	24a	Provide registration information for the review, including register name and registration number, or state that the review was not registered.	5
	24b	Indicate where the review protocol can be accessed, or state that a protocol was not prepared.	5
	24c	Describe and explain any amendments to information provided at registration or in the protocol.	5
Support	25	Describe sources of financial or non-financial support for the review, and the role of the funders or sponsors in the review.	20
Competing interests	26	Declare any competing interests of review authors.	20
Availability of data, code and other materials	27	Report which of the following are publicly available and where they can be found: template data collection forms; data extracted from included studies; data used for all analyses; analytic code; any other materials used in the review.	20

The current checklist followed the latest PRISMA 2020 guideline. [28]

Table S2. Keyword and search results in each database.

Database	Keyword	Filter	Date	Result
PubMed	(long COVID-19 syndrome) AND (hearing loss OR sensorineural hearing loss OR SNHL OR audiology OR tinnitus OR vertigo OR vestibular OR dizziness)	N/A	2025/12/15	104
Embase	(long COVID-19 syndrome) AND (hearing loss OR sensorineural hearing loss OR SNHL OR audiology OR tinnitus OR vertigo OR vestibular OR dizziness)	N/A	2025/12/15	949
ClinicalKey	(long COVID-19 syndrome) AND (hearing loss OR sensorineural hearing loss OR SNHL OR audiology OR tinnitus OR vertigo OR vestibular OR dizziness)	N/A	2025/12/15	170
Web of Science	(long COVID-19 syndrome) AND (hearing loss OR sensorineural hearing loss OR SNHL OR audiology OR tinnitus OR vertigo OR vestibular OR dizziness)	N/A	2025/12/15	93
ScienceDirect	(long COVID-19 syndrome) AND (hearing loss OR sensorineural hearing loss OR SNHL OR audiology OR tinnitus OR vertigo OR vestibular OR dizziness)	N/A	2025/12/15	4883

Abbreviation: N/A: not applied.

Table S3. Excluded studies and reason.

Reason	Numbers	References
Meta-analysis not related to long COVID-19 syndrome	2	[6,87]
Not related to audiovestibular dysfunction	3	[10,11,14]
Research not related to long COVID-19 syndrome	5	[2,7,8,20,24]
Review article not related to long COVID-19 syndrome	5	[89–93]

Table S4. Newcastle-Ottawa Scale and Characteristics for the Included Trial (observational trial).

Study	**Selection**				**Comparability**	**Exposure**			Total
	Case definition	Representative	Control selection	Control definition	Comparability	Ascertainment	Same method	Non-Response rate	Summary
Niemczak, C. (2025) [44]	*	*	*	*	*	*	*		7*
Yamashita, L.D. (2025) [34]	*	*				*		*	4*
Chen, E.Y. (2024) [39]	*	*				*		*	4*
Hamdy, M.M. (2024) [45]	*	*	*	*	*	*	*		7*
Kelleni, M.T. (2024) [60]	*	*				*			3*
Noij, K.S. (2024) [62]	*	*				*		*	4*
Dorobisz, K. (2023) [54]	*	*	*	*	*	*	*		7*
Hastie, C.E. (2023) [68]	*	*	*	*	*	*	*		7*
Marinkovic, K. (2023) [47]	*	*	*	*	*	*	*		7*
Obeidat, F.S. (2023) [67]	*	*				*		*	4*
Rios Coronado, O.O. (2023) [35]	*	*				*		*	4*
Saniasiaya, J. (2023) [37]	*	*				*			3*
Almishaal, A.A. (2022) [23]	*	*	*	*	*	*	*		7*

Bhatta, S. (2022) [31]	*	*	*	*	*	*	*		7*
Boboshko, M.Y. (2022) [26]	*	*				*		*	4*
Degen, C.V. (2022) [33]	*	*				*		*	4*
Erinc, M. (2022) [66]	*	*	*	*	*	*	*		7*
Figueiredo, R.R. (2022) [56]	*	*	*	*	*	*	*		7*
Gervasoni, F. (2022) [63]	*	*				*		*	4*
Kokten, N. (2022) [52]	*	*				*		*	4*
Ludwig, S. (2022) [38]	*	*				*		*	4*
Ozturk, B. (2022) [53]	*	*	*	*	*	*	*		7*
Pazdro-Zastawny, K. (2022) [36]	*	*				*		*	4*
Yildiz, E. (2022) [51]	*	*	*	*	*	*	*		7*
Yilmaz, O. (2022) [55]	*	*	*	*	*	*	*		7*
Gedik, O. (2021) [21]	*	*	*	*	*	*	*		7*
Meinhardt, J. (2021) [46]	*	*	*	*	*	*	*		7*
Versace, V. (2021) [50]	*	*	*	*	*	*	*		7*
Koumpa, F.S. (2020) [58]	*	*				*			3*

Lang, B. (2020) [59]	*	*	*	3*
Reichard, R.R. (2020) [48]	*	*	*	3*

* indicated this study have a good performance in this item.

Table S5. Summary of the included study.

Study	Characteristics		Outcomes	Summary
	Study design	Recruited case characteristics	Results	Conclusion
Niemczak, C. (2025) [44]	Prospective case-control study	Eighty-two individuals participated in this cross-sectional study, including 37 with Post-Acute Sequelae of SARS-CoV-2 Infection (mean age: 47.5, Female: 83%) and 45 healthy controls (mean age: 38.5, Female: 76%)	The increase in latency correlated with cognitive fatigue scores and predicted PASC status. The ABR V/I amplitude ratio was examined as a measure of central gain. Although these ratios were not significantly elevated in the full PASC group, to minimize the cofounding effect of age, the cohort was median split on age. Elevated V/I amplitude ratios were significant predictors of both predicted PASC group classification and cognitive fatigue scores in the younger PASC subjects compared to age-matched controls providing evidence of elevated central gain in younger individuals with PASC	More frequent tinnitus also significantly predicted higher subjective cognitive fatigue scores. Our findings suggest that PASC may alter the central auditory pathway and lead to slower conduction and elevated auditory neurophysiology responses at the midbrain, a pattern associated with the typical aging process. This study marks a significant stride toward establishing an objective measure of subjective cognitive fatigue through assessment of the central auditory system
Yamashita, L.D. (2025) [34]	Retrospective cross-sectional study	Participants include 240 patients with documented evidence of a positive SARS-CoV-2 PCR or antibody test who underwent initial evaluation and 182 patients with longitudinal follow-up	Severe infection, fewer years of education level, and non-White race were found to be statistically associated with an increased likelihood of having abnormal scores on cognitive testing.	Performance on standardized cognitive screening tests may not be consistent with frequently reported cognitive complaints in Long COVID patients. The most common clinical trajectory was self-reported improvement in the primary neurological symptom.
Chen, E.Y. (2024) [39]	Retrospective cross-sectional study	Pediatric patients with long COVID-19 syndrome	Preexisting mood disorders were associated with a higher prevalence of worsening mental health symptoms (anxiety, P = 0.01; depression, P = 0.04),	Long COVID has a significant impact on the quality of life of children and their families. Children with long COVID can

			dizziness/lightheadedness/vertigo (P = 0.02), and change in appetite (P = 0.04).	benefit from multidisciplinary care addressing fatigue, mental health, and family coping.
Hamdy, M.M. (2024) [45]	Prospective case-control study	Forty post-recovery patients who tested positive for SARS-CoV-2 made up the study group.	ABR recording for both ears showed significant difference between cases and controls as regarding absolute latencies of wave I, III, V, wave V (high rate), amplitude (low and high rates), amplitude ratio, and interaural latency differences (III, I-III, I-V), while P300 outcomes showed a statistically significant difference between cases and controls regarding P300 latency ($p < 0.05$), while for amplitude, a highly significant difference was found.	COVID-19 can harm both the inner ear and the auditory pathway, and it has long-lasting effects on the auditory system and on cognitive processing and attention.
Kelleni, M.T. (2024) [60]	Case report	In this short communication, the author discusses how he suffered from idiopathic tinnitus and how he managed to adopt a combined pathophysiological and pharmacological approach to the reason for the first time in the medical literature that low-dose metformin might be safely and effectively repurposed to manage at least a subset of tinnitus patients while discussing the potential role of adenosine receptor agonists as potential future tinnitus therapeutics.	This is the first academic call to repurpose a low- dose 500 mg metformin q.d. to manage tinnitus and it is reasonable to suggest that at least some tinnitus patients, using metformin 500 mg q.d., might appreciate the calm muffling of its sharp bothersome ringing tone	Finally, I suggest that physicians who manage patients in developing countries where the most modern methods used for its management, though largely futile, are neither available nor affordable, as well as those who manage frustrated patients in developed countries who are suffering the lack of an effective solution, should consider our current call and I suggest that muffling, not complete absence, of tinnitus should be the parameter to inquire about in any tinnitus clinical trial.
Noij, K.S. (2024) [62]	Cross-sectional study	A series of 9 adolescent patients were referred from a multidisciplinary long-COVID clinic and diagnosed with PPPD.	Recommended treatment included vestibular physical therapy, selective serotonin reuptake inhibitor medication, and cognitive behavioral therapy. The majority of patients experienced an improvement in their symptoms, and all patients had improved activity levels and DHI-pc scores after treatment.	No previous reports exist discussing PPPD in long-COVID patients. This case series provides insight into symptom evolution and treatment efficacy in this patient population.

Dorobisz, K. (2023) [54]	Case-control study	The study group included 58 patients aged 23 to 75 years who were diagnosed with COVID-19 infection six months before inclusion in the present study and reported post-COVID-19 hearing impairment.	There were statistically significant differences between the control and study groups. Sensorineural hearing loss was found in 65.5% of the tonal audiometry test. The stapes reflex was absent in almost 20% of post-COVID-19 patients. The analysis of ABRs demonstrated longer latencies of wave III, V, and time intervals I-III, I-V in post-COVID-19 patients.	COVID-19 can damage the inner ear as well as the auditory pathway. Hearing loss may be the only symptom of COVID-19 or be a late complication of the disease due to postinfectious inflammation of the nerve tissue as a symptom of long COVID-19. Prolonged conduction of the auditory pathway shows the affinity of the virus to the nervous system as a symptom of long COVID. It is advisable to perform hearing diagnostics in patients after COVID-19 and provide them with specialist care.
Hastie, C.E. (2023) [68]	Prospective case-control study	Every adult (>16 years) in Scotland with a positive PCR test from April 2020 was invited along with a comparison group who had had a negative test but never a positive test (hereafter referred to as never infected), matched by age, sex, deprivation quintile, and time period of test	Of those with previous symptomatic infection, 35% reported persistent incomplete/no recovery, 12% improvement and 12% deterioration. At six and 12 months, one or more symptom was reported by 71.5% and 70.7% respectively of those previously infected, compared with 53.5% and 56.5% of those never infected.	Altered taste, smell and confusion improved over time compared to the never infected group and adjusted for confounders. Conversely, late onset dry and productive cough, and hearing problems were more likely following SARS-CoV-2 infection.
Marinkovic, K. (2023) [47]	Prospective case-control study	Previously healthy adults (24.4 ± 5.2 years of age) who experienced PASC for almost 6 months following a mild acute COVID-19 illness	Controlling for tissue composition, biological sex, and alcohol intake, the PASC group had lower GABA+/water than CNT, which correlated with depression and poor sleep quality. The mediation analysis revealed that the impact of PASC on depression was partly mediated by lower GABA+/water, indicative of cortical hyperexcitability as an underlying mechanism. In addition, N-acetylaspartate (NAA) tended to be lower in the PASC group, possibly suggesting compromised neuronal integrity.	Persistent neuroinflammation may contribute to the pathogenesis of PASC-related neurocognitive dysfunction.

Obeidat, F.S. (2023) [67]	Cross-sectional study	People aged between 18 and 60 years old who had had COVID-19 at least 8 weeks before the beginning of the study were included.	After controlling for age and severity of dizziness, female sex and high fatigue severity were associated with an increased likelihood of reporting dizziness (R^2 = 31%). The severity of dizziness and neurological symptoms during the acute stage of COVID-19 were associated with an increased likelihood of reporting hearing loss (R^2 = 10.4%) after controlling for age.	Dizziness and hearing loss present in long COVID and can be disabling. Females with high levels of fatigue should be questioned about persistent dizziness. Hearing loss should be considered in individuals with neurological symptoms and severe dizziness as a consequence of long COVID.
Rios Coronado, O.O. (2023) [35]	Cross-sectional study	Patients with a diagnosis of COVID-19 infection was included: The age range was between 18 and 50 years old, the mean age was 37.0 years with a standard deviation of ±8.3 years, and 32 patients (68.1%) were female and 15 male patients (31.9%)	The audiological symptoms presented a prevalence of 74.4% for a sensation of ear fullness, 59.6% for tinnitus, and 51.1% for a sensation of hearing loss.	Audiological research can help identify risk factors and symptoms associated with auditory complications, enabling healthcare providers to initiate timely interventions and reduce the burden of long-term auditory problems. This is the initial stage of comprehensive research that will thoroughly analyze variables researched within this study such as tinnitus, over a prolonged period.
Saniasiaya, J. (2023) [37]	Case report	Two cases of vestibular migraine post-COVID-19 involving two children who presented with vestibular migraine symptoms following COVID-19 infection	After a subsequent review in 1 month, the child claims his symptoms were well controlled with tablet flunarizine. He only required additional analgesics during two episodes, which resolved after 15 min of oral analgesic. The patient claims his quality of life has improved dramatically. He was advised to continue taking tablet flunarizine for another 3 months prior to his review.	Children post-COVID-19 should be thoroughly evaluated for vestibular migraine symptoms so they can be managed promptly. This is the first article to report vestibular migraine as a manifestation of long COVID-19 syndrome
Almishaal, A.A. (2022) [23]	Cross-sectional study	Severe hospitalized cases and nonhospitalized patients with mild disease, all with confirmed SARS-CoV-2 test results	Auditory symptoms were reported by 21.9% and 1.99% of patients during the acute phase and 6 months post SARS-CoV-2 infection, respectively. During the acute phase of SARS-CoV-2 infection, aural fullness represents the most common symptoms (18.94%) followed by tinnitus (9.97%) and hearing loss (6.31%). Vestibular symptoms were reported by 34% during the acute phase; most commonly	The current study showed that audiovestibular symptoms are common among SARS-CoV-2 infected patients during the acute phase of the disease. However, these symptoms are mostly temporary and showed complete spontaneous recovery during the first 2 weeks postinfection

			was dizziness (29.9%) followed by vertigo (24.25%) and unsteadiness (8.31%)	
Bhatta, S. (2022) [31]	Prospective case-control study	The patients positive for the COVID-19 infection as confirmed by the reverse transcriptase polymerase chain reaction	Aural symptoms were, tinnitus in 1.8%, aural fullness in 1.4%, hearing loss in 3. 9%, and ear ache in 1.8% were present initially, resolved at 3 months follow up. The impedance audiometry demonstrated type B and type C curve in 5.1% and 1.15% ears, and out of these 64.7% and 40% improved at 3 months follow up respectively	The COVID-19 infection may present with aural symptoms; however, it was concluded that there was no significant difference in the hearing status of the COVID-19 positive patients in comparison to the control group. The presence of some changes in the normal functioning of the eustachian tube and middle ear in the COVID-19 infection was also highlighted.
Boboshko, M.Y. (2022) [26]	Cross-sectional study	Patients age above 18 years, complaints of hearing impairment and/or tinnitus during or after COVID-19, and recovery from COVID-19 no earlier than 2 weeks and no later than 24 weeks before the audiological check-up.	A total of 81% of patients complained about hearing disorders, and 43% noted memory impairment. According to pure tone audiometry, 24% of the subjects had normal hearing, while 76% had some degree of hearing loss. No significant changes in hearing thresholds were found in comparison with audiological examinations performed before COVID-19. Disorder of monosyllabic words' intelligibility in quiet was found in 33% of patients, and in 42% in noise, along with low indicators in the dichotic digits test in 54% of patients. Moreover, 71% of patients had low scores on the MoCA scale that indicated cognitive impairment.	The deterioration of speech test scores in patients after COVID-19 can occur due to central auditory processing disorders (CAPD), memory impairment, or changes in cognitive status in general.
Degen, C.V. (2022) [33]	Cross-sectional online-survey study	Participants who were at least 18 years old with symptoms more than 4 weeks after a SARS-CoV-2 infection, confirmed by a self-reported positive PCR, antibody or antigen test, were included in the study	At the time of the survey, 60% of patients reported the presence of vertigo or dizziness with a mean severity of 4.6 ± 2.7 on a scale of 1 (least severe) to 10 (most severe) and 30% complained of tinnitus with a mean severity of 4.8 ± 3.0	The self-reported severity highlights the need for Long COVID clinics to address these symptoms effectively
Erinc, M. (2022) [66]	Case-control study	All patients were diagnosed with either bilateral or unilateral	There was no decrease in the tinnitus complaint during COVID-19, 24.3% of the infected patients reported	This study points to possible different effects of the infection with SARS-CoV-2

		chronic idiopathic tinnitus, and 75% had no comorbid disease	exacerbation of tinnitus, and 75.7% said tinnitus remained the same. In the COVID-19-negative group, 13.5% reported tinnitus decrease during the pandemic, 57.6% said it remained the same, and 28.8% reported exacerbation of tinnitus.	and the pandemic period on patients with chronic tinnitus. It also provides evidence for deterioration of preexisting tinnitus as a possible long-term effect of COVID-19.
Fancello, V. (2022) [61]	Review article	Not applicable	The course of COVID-19 infection may be complicated by a variety of neurological manifestations. Since the inner ear is vulnerable to viruses, sensorineural hearing loss (SNHL) has been reported to occur following the SARS-CoV-2 infection, often resulting in long-term morbidity and worsening the quality of life. The interest in how the virus affects the inner ear has gradually increased since the pandemic's spread, but little is still known about the SNHL potentially caused by SARS-CoV-2. The aim of this paper is to evaluate the possible association between SNHL and COVID-19 infection, through a systematic literature review.	Currently available data suggest that SARS-CoV-2 may hamper cochlear function; however, available reports are still limited. Large cohort and prospective studies are necessary to evaluate the long-term effects of this viral infection in the inner ear.
Figueiredo, R.R. (2022) [56]	Case-control study	Fifty-seven patients over 18 years old and previously diagnosed with COVID-19 confirmed by a RT-PCR test were included.	PCT was reported by 19.3% of the patients, while 22.8% reported CT. No statistical difference was found between CT and PCT concerning hearing function, tinnitus characteristics and tinnitus distress. There was also no statistically significant difference between PCT and NT with respect to COVID-19 symptoms and pharmacological COVID-19 treatment. Patients with CT reported worsening of their tinnitus after COVID-19.	Among those patients who had tinnitus before COVID-19 30,8% reported worsening after COVID-19. Overall, tinnitus emerging in the context of a COVID-19 infection seems not to differ from tinnitus unrelated to COVID-19. For further exploring the relationship of tinnitus and COVID-19, large population based studies are warranted.
Gervasoni, F. (2022) [63]	Cross-sectional study	Post-COVID-19 outpatients (mean age 47.3 ± 11.1 years, 50 females, 25 hospitalized), evaluated using the robotic device hunova	These results suggest that independently from the severity of the disease experienced, post-COVID syndrome makes the elastic balance test performances more distant from the normality when the subject integrates vision, somatosensory information, and vestibular information. In the absence of visual feedback, patients seem to implement compensatory strategies, presumably seeking	These data suggest a new mechanism of the post-COVID syndrome that deserves further investigation for its potential impact on activities of daily living.

			more significant feedback from the lower limbs, which improve their performance	
Kokten, N. (2022) [52]	Cross-sectional study	Thirty healthcare workers who had COVID-19 after hearing evaluation with pure tone audiometry (PTA) for any reason in the last 1 year were included in the study.	When the PTA results of 30 patients (60 ears) before and after COVID-19 were compared, a significant decrease in hearing level was found only at 1000 Hz ($p < .05$). There were no significant differences at other frequencies. When the PTA and TEAOE test results of 15 patients (30 ears) that were performed during and after COVID-19 were compared, no significant differences were found.	We conclude that COVID-19 may cause hearing loss. However, this result needs to be confirmed with comprehensive studies to be conducted in larger patient groups.
Ludwig, S. (2022) [38]	Cross-sectional study	Adulthood (>18 years), being six months post-diagnosis of COVID-19, as confirmed by polymerase chain reaction (PCR) from swab samples, and approval by the local health offices.	After COVID-19, clinical tests revealed that 75% were suffering from hyposomnia/anosmia, and 20% of all patients reported mild hypogeusia for up to six months	COVID-19 particularly caused olfactory and gustatory impairment; balance disorders were present too; vestibular and auditory symptoms were negligible.
Ozturk, B. (2022) [53]	Case-control study	Thirty individuals between the ages of 18-45, who were diagnosed with COVID-19 by PCR at least one month ago, and had no pre-COVID-19 hearing loss complaints, constituted the test group	A significant difference was found between the groups at all high frequencies between 4 and 14 kHz ($p < 0.05$). TEOAE amplitudes at 1500 Hz, 2000 Hz and 4000 Hz frequencies and DPOAE amplitudes at 4003 Hz and higher frequencies were significantly lower in the test group ($p < 0.05$). While there was a significant difference between the I, III and V absolute latencies between the groups ($p < 0.05$), there was no significant difference between the I-III, III-V and I-V interpeak latencies ($p > 0.05$) as a result of the ABR test.	This study showed that COVID-19 can cause cochlear damage, especially at high frequencies. More studies are needed to determine the effects of COVID-19 on the auditory system.
Pazdro-Zastawny, K. (2022) [36]	Cross-sectional study	Patients aged 23-75 years with vertigo, who were diagnosed with COVID-19 infection 6 months before the examination.	Positional nystagmus was observed in 15 patients (24.1%). Asymmetrical optokinetic nystagmus was observed in 18 patients (31%). A distorted record in the tracking pendulum test was present in 23 patients (39.7%). Square waves were observed in 34 COVID-19 patients (58.6%).	Patients who had been diagnosed with COVID-19 seem to be more likely to suffer from vertigo/dizziness and to compensate more slowly. COVID-19 infection may cause inner ear damage and lead to vestibular dysfunction. The role of the central nervous system in the

				onset of equilibrium disorders should be considered.
Yildiz, E. (2022) [51]	Case-control study	Patients with COVID-19 disease and COVID-19 pneumonia, and control group patients.	PTA results and TEOAE amplitudes in the first and third months were not significantly different between the COVID-19 non-pneumonia group and the control group (p > 0.05), between the COVID-19 pneumonia group and the control group (p > 0.05), and between the COVID-19 non-pneumonia group and the COVID-19 pneumonia group (p > 0.05).	Despite minimal impairment and minimal amplitude decreases in patients, who recovered from COVID-19, such changes were found to become restored in the third month. Furthermore, no significant changes were observed to indicate COVID-19- associated hearing loss.
Yilmaz, O. (2022) [55]	Case-control study	Thirty-seven patients who recovered from Covid-19 disease and 30 healthy controls were compared using Dizziness Handicap Inventory (DHI), Computerized Dynamic Posturography (CDP), Vestibular Evoked Myogenic Potentials(VEMP) and Video Head Impulse Test (v-HIT).	On CDP, the composite and visual general scores of the patients were significantly lower than controls (p<0.01). The v-HIT gains of the patients significantly decreased in the vertical semicircular canals compared to controls (p<0.01).There was a significant difference between the patients and controls regarding the absence of o-VEMPs (p<0.01) while the amplitudes and latencies were similar between the groups (p>0.05). Decreased P1/N1 amplitudes and elongated N1 latencies were found on c-VEMP testing (p<0.05). Anosmia, taste disorder and gender were not associated with subjective and objective test results (p>0.05).	The Covid-19 disease can cause dizziness rather that incapacitating vertigo. Dizziness can be seen in almost one-fifth of the adult covid19 out-patients, which may be due to involvement of vestibular and visual systems,ortheir central connections. The squeals created in the balance related systems may be irreversible as they have persisted after the recovery of the patients. It is also plausible to anticipate more severe condition in the older patients who were treated in the intensive care units. In the long term follow up of the survivors, the need for balance rehabilitation programs should be remembered in order to minimize risks of falling down.
De Luca, P. (2021) [43]	Review article	Not applicable	Despite rarely, auditory impairment can appear in patients with COVID-19 and should always be investigated for an early treatment and potential indicator of involvement of the central nervous system	Hearing loss, despite rarely, might be present in COVID-19 patients. Auditory evaluation, although with all preventive measures to prevent contagion for healthcare providers, should be performed, especially if hearing

				disturbance are self-reported. The early recognition of these non-specific symptoms, which might be an early sign of brain inflammation, could help in preventing the spread of the infection to other areas of the brain.
Gedik, O. (2021) [21]	Case-control study	Twenty individuals with no history of coronavirus disease 2019 and 27 individuals diagnosed with coronavirus disease 2019 were compared	The pure tone audiometry and extended high-frequency mean threshold values were higher in the coronavirus disease 2019 group. The transient evoked otoacoustic emissions signal-to-noise ratios were bilaterally lower at 4 kHz in individuals with a coronavirus disease 2019 history. In the auditory brainstem response test, only the interpeak latencies of waves III-V were significantly different between groups.	Coronavirus disease 2019 may cause damage to the hearing system. Patients should be followed up in the long term with advanced audiological evaluation methods in order to determine the extent and level of damage.
Lopez-Leon, S. (2021) [32]	Meta-analysis	Patients at a post-COVID-19 stage (assessed 2 weeks or more after initial symptoms) in cohorts of COVID-19 patients	The five most common symptoms were fatigue (58%), headache (44%), attention disorder (27%), hair loss (25%), and dyspnea (24%)	Multi-disciplinary teams are crucial to developing preventive measures, rehabilitation techniques, and clinical management strategies with whole-patient perspectives designed to address long COVID-19 care
Meinhardt, J. (2021) [46]	Tissue case-control study	Thirty-three deceased individuals with COVID-19 either confirmed by PCR for SARS-CoV-2 (*n* = 31 of 33) or with clinical features highly suggestive of COVID-19 (*n* = 2 of 33) were included	By various means, we demonstrate the presence of SARS-CoV-2 RNA and protein in anatomically distinct regions of the nasopharynx and brain. Furthermore, we describe the morphological changes associated with infection such as thromboembolic ischemic infarction of the CNS and present evidence of SARS-CoV-2 neurotropism. SARS-CoV-2 can enter the nervous system by crossing the neural-mucosal interface in olfactory mucosa, exploiting the close vicinity of olfactory mucosal, endothelial and nervous tissue, including delicate olfactory and sensory nerve endings.	Subsequently, SARS-CoV-2 appears to follow neuroanatomical structures, penetrating defined neuroanatomical areas including the primary respiratory and cardiovascular control center in the medulla oblongata.
Versace, V. (2021) [50]	Case-control study	Twelve patients who recovered from typical COVID-19	Post-COVID-19 patients reported marked fatigue according to FRS score (8.1 ± 1.7) and presented pathological scores	The present study documents for the first time reduced GABAergic inhibition in the

		pneumonia with neurological complications and complained of profound physical and mental fatigue	at the FAB based on Italian normative data (12.2 ± 0.7). TMS revealed marked reduction of SICI, and disruption of LICI as compared to HS. SAI was also slightly diminished.	M1 in patients who recovered from COVID-19 with neurological complications and manifested fatigue and dysexecutive syndrome.
Yong, S.J. (2021) [49]	Review article	Not applicable	Long-COVID is a postviral illness that can affect survivors of COVID-19, regardless of initial disease severity or age. Symptoms of long-COVID include fatigue, dyspnea, gastrointestinal and cardiac problems, cognitive impairments, myalgia, and others. While the possible causes of long-COVID include long-term tissue damage, viral persistence, and chronic inflammation, the review proposes, perhaps for the first time, that persistent brainstem dysfunction may also be involved.	As neurons do not readily regenerate, brainstem dysfunction may be long-lasting and, thus, is long-COVID. Indeed, brainstem dysfunction has been implicated in other similar disorders, such as chronic pain and migraine and myalgic encephalomyelitis or chronic fatigue syndrome.
Angileri, F. (2020) [42]	Review article	Not applicable	Molecular mimicry has been proposed as a cause of the autoimmune phenomena observed in COVID-19, the syndrome associated with the infection by severe acute respiratory syndrome coronavirus 2 (SARS-CoV-2). Lucchese and Flöel [4] have recently reported three human proteins (namely DAB1, AIFM, and SURF1, as catalogued at www.uniprot.org) – that are present in neurons of the respiratory pacemaker in the brainstem – that share potentially antigenic epitopes with SARS-CoV-2, as shown by in silico analysis.	Although plasma membrane localization has been demonstrated only for OR7D4 and SLC12A6, we cannot exclude that, after cell stress, post-translational modifications could induce PARP9 trafficking to plasma membrane and its exposure on the cell surface, as for other intracellular proteins. Other studies, including the analysis of anatomical specimens from autopsies of subjects who died from severe forms of COVID-19, are necessary to verify these predictions.
Davies, J. (2020) [40]	Protein database study	Not applicable	It is now well-established that entry of SARS-CoV-2 into host cells is facilitated by its spike proteins mainly through binding to the angiotensin-converting enzyme 2 (ACE-2). Preclinical studies have suggested that neuropilin-1 (NRP1), which is a transmembrane receptor that lacks a cytosolic protein kinase domain and exhibits high expression in the respiratory and olfactory epithelium, may also be implicated in COVID-19 by enhancing the entry of	This furthers supports the potential role of NRP1 as an additional SARS-CoV-2 infection mediator implicated in the neurologic manifestations of COVID-19. Accordingly, the neurotropism of SARS-CoV-2 via NRP1-expressing cells in the CNS merits further investigation.

			SARS-CoV-2 into the brain through the olfactory epithelium. In the present study, we expand on these findings and demonstrate that the NRP1 is also expressed in the CNS, including olfactory-related regions such as the olfactory tubercles and paraolfactory gyri.	
Greenhalgh, T. (2020) [57]	Review article	Not applicable	Around 10% of patients who have tested positive for SARS-CoV-2 virus remain unwell beyond three weeks, and a smaller proportion for months. This is based on the UK COVID Symptom Study, in which people enter their ongoing symptoms on a smartphone app. This percentage is lower than that cited in many published observational studies, whose denominator populations were those admitted to hospital or attending specialist clinics. A recent US study found that only 65% of people had returned to their previous level of health 14-21 days after a positive test.	Patients, many of whom were young and fit before their illness, have described being dismissed or treated as hypochondriacs by health professionals. They have rightly contested the classification of non-hospitalised covid-19 as “mild.” In these uncertain times, one key role that the primary care practitioner can play is that of witness, “honouring the story” of the patient whose protracted recovery was unexpected, alarming, and does not make sense
Hu, J. (2020) [41]	Review article	Not applicable	A novel coronavirus (SARS-CoV-2) emerged from Wuhan, China, and spread quickly around the world. In addition to fever, cough and shortness of breath, it was confirmed that the patients also have manifestations towards the central nervous system (CNS), especially those critically ill ones. In this review, we will discuss how SARS-CoV-2 gain access to the CNS and the possible consequences. Both SARS-CoV-2 and SARS-CoV-1 in 2002 share the same receptor angiotensin-converting enzyme 2 (ACE2), which can be found in the brain and mediate the disease process.	Both direct attack of SARS-CoV-2 and the abnormal immune response in the CNS would contribute to the disease. Also, there is a relationship between SARS-CoV-2 and the occurrence of acute cerebrovascular diseases.
Koumpa, F.S. (2020) [58]	Case report	A case of sudden onset sensorineural hearing loss following COVID-19	Physical examination and imaging excluded any other cause of hearing loss. A literature review showed that four other cases have been previously described. Hearing loss can be a significant cause of morbidity and can easily be missed in the intensive care setting.	Being aware and screening for SSNHL following COVID-19 enables an early course of steroids, which offers the best chance of recovering hearing.

Lang, B. (2020) [59]	Case report	A case of a 30-year-old female staff nurse who contracted coronavirus disease 2019 and presented to our department with a significant unilateral sensorineural hearing loss confirmed on audiogram.	She was treated with a course of oral steroids, but unfortunately there was no improvement in her hearing.	This case report is important as it highlights the importance of having a low index of suspicion when patients present with a variety of symptoms not previously associated with coronavirus disease 2019.
Reichard, R.R. (2020) [48]	Case report	Neuropathological findings of a patient who died from complications of COVID-19	The autopsy revealed a range of neuropathological lesions, with features resembling both vascular and demyelinating etiologies. Hemorrhagic white matter lesions were present throughout the cerebral hemispheres with surrounding axonal injury and macrophages. The subcortical white matter had scattered clusters of macrophages, a range of associated axonal injury, and a perivascular acute disseminated encephalomyelitis (ADEM)-like appearance.	The clinical course of the patient also illustrates that during prolonged hospitalizations neurological complications of COVID may develop, which are particularly difficult to evaluate and appreciate in the critically ill.
